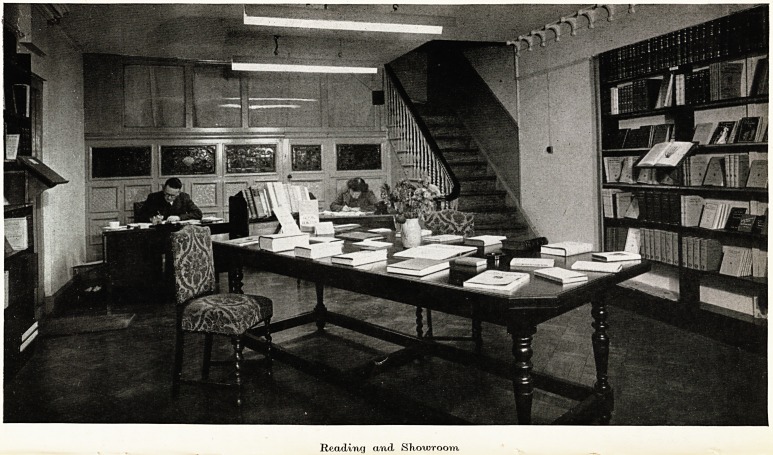# House of John Wright

**Published:** 1947

**Authors:** 


### HOUSE OF JOHN WRIGHT

(JOHN WRIGHT & SONS LIMITED)

*Medical Printers and Publishers*

ON October 1st, 1947, a new medical bookshop and reading room was opened at 42–44 The Triangle, by John Wright & Sons Ltd. Stocks are held of the works of most medical publishers and any book not in stock can be ordered. It is hoped that doctors and medical students will make full use of the room for study and reference. They will find a quiet and dignified atmosphere quite unlike that of the ordinary bookshop.

John Wright & Sons Ltd. were established as printers in 1825 and as publishers soon afterwards. The founder's main interest was in religious and educational literature and the firm first turned to medical work in 1885 when the publishing rights in the *Medical Annual*, then small and unsuccessful, were acquired in part settlement of a bad debt. The Company was then under the chairmanship of the son of the founder, Mr. Hartland S. Wright, and from that date the medical side of the business has been steadily expanded. It is, however, interesting to note that a pamphlet entitled "Physiological Facts connected with the Function of Respiration," published by the firm in 1836, has recently come to light in the library of the B.R.I.

The Company is associated in the minds of most Bristolians with the Colston Avenue building, Stonebridge House, where the printing and publishing offices stood from 1881–1941. Even during this period, in 1905, the premises were destroyed by fire but in those piping days of peace reconstruction did not take long and the opportunity was taken to modernize the works and to absorb the adjacent premises. In 1941 Stonebridge House was completely destroyed in the blitz with the loss of the lives of two fire watchers. All but three machines were lost and there was enormous destruction of book stocks and damage to standing matter. Reconstruction on the site was impossible but premises were found at Weston-super-Mare and for a year Wright's medical publications were largely produced there. Here it was possible to do something to replace the losses suffered at Bristol, but in 1942 these new premises too were blitzed, and in those desperate days it was found that another start was out of the question. After the first blitz in 1941 publishing offices had been found in Orchard Street and from here the Company carried on, the book printing now being executed by a number of other printers. With the end of hostilities these offices became too

*A corner of Boardroom with Chairman*

Reading and Showroom small and in 1947 new ones were taken over at 42–44 The Triangle, where the medical bookroom referred to above was opened.

Since its inception in 1913 the *British Journal of Surgery*, of which our late colleague Professor Hey Groves was the Editorial Secretary, has been printed and published by Wrights of Bristol. The production of the *Journal* was uninterrupted by the war in spite of the destruction of premises and loss of matter in type, and as a mark of appreciation the Editorial Board presented the publishers with a carved oak tablet with the following inscription:—

> "This tablet presented by the Editorial Committee records that in spite of repeated enemy action John Wright and Sons Ltd. maintained the regular production and publication of the *British Journal of Surgery* throughout the World War 1939–45.
>
> GEORGE E. GASK, Chairman."

It truly was a remarkable performance which deserved this signal recognition.

Even before the war was over, realizing that the fate of the old site would remain uncertain for a long time, the Company acquired a new site at Brislington and plans were drawn up for a modern printing works. Progress has been unavoidably slow but it will soon be possible to go into production here. The new works will be known as The Stonebridge Press, to perpetuate the name of the old premises, and the name and descriptive colophon have no doubt been noticed on the latest copies of the firm's publications. The opportunity has been taken to plan this factory on modern lines and nearly all the machinery will be new. Nothing will therefore stand in the way of production of the finest quality, which has always been the aim of the Company, their colour illustrations especially being renowned all the world over. From its earliest days the firm has been in the forefront of technical developments—they introduced the first book printing machine and steam driven press in the West of England, and the first book folding machine and Monotype in Bristol—and the same progressive outlook will be maintained in the new era upon which the Company is about to enter.

It is probably little realized how large a part medical publishing plays in the export drive, for over 50 per cent. of the output goes abroad. Perhaps medical men and women in this country will remember this when they find difficulty and delay in obtaining the works they need! Many of the books of John Wright & Sons Ltd. are translated into foreign languages and the translation rights also form a valuable invisible export.

The Company is under the direction of Mr. John Wright, the

[*Continued on page* 115

### HOUSE OF JOHN WRIGHT

(*Continued from page* 107)

grandson of the founder, as Chairman of the Board, assisted by his son, Mr. Philip Wright, with Dr. F. S. Hunter and Mr. L. G. Owens on the Editorial side of the business. In the past and in the present Wrights of Bristol have been able to play their part in serving the medical profession at home and abroad, and in the future, when the Stonebridge Press is in full production, we feel confident that under this experienced direction they will be able to do so to an even greater extent.

## Figures and Tables

**Figure f1:**
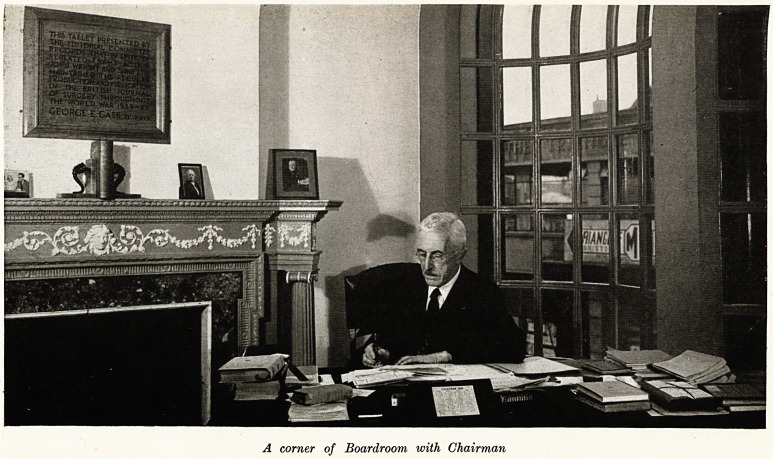


**Figure f2:**